# Strengths and weaknesses of parent–staff communication in the NICU: a survey assessment

**DOI:** 10.1186/1471-2431-13-71

**Published:** 2013-05-07

**Authors:** Helena Wigert, Michaela Blom Dellenmark, Kristina Bry

**Affiliations:** 1 Institute of Health and Care Sciences, The Sahlgrenska Academy at University of Gothenburg, Box 457, Gothenburg, SE 405 30, Sweden; 2 Division of Neonatology, Sahlgrenska University Hospital, Gothenburg, 416 85, Sweden; 3 Division of Pediatric Emergency Care and Pediatric Surgery, Sahlgrenska University Hospital, Gothenburg, 416 85, Sweden; 4 Department of Pediatrics, The Sahlgrenska Academy at University of Gothenburg, Gothenburg, 416 85, Sweden

**Keywords:** Parents’ experiences, Communication, Neonatal intensive care unit, Survey assessment

## Abstract

**Background:**

Parents of infants hospitalized in the neonatal intensive care unit (NICU) find themselves in a situation of emotional strain. Communication in the NICU presents special challenges due to parental stress and the complexity of the highly technologized environment. Parents’ need for communication may not always be met by the NICU staff. This study aimed to describe strengths and weaknesses of parent–nurse and parent–doctor communication in a large level III NICU in Sweden in order to improve our understanding of parents’ communication needs.

**Methods:**

Parents were asked to complete a survey consisting of sixteen questions about their experiences of communication with nurses and doctors in the NICU. In each question the parents evaluated some aspect of communication on a five- or six-point Likert scale. They also had the opportunity on each question to comment on their experiences in their own words. Data were analyzed using IBM SPSS Statistics 20.0 and qualitative manifest content analysis.

**Results:**

270 parents (71.4%) completed the survey. Parents generally rated communication with the staff in the NICU positively and appreciated having received emotional support and regular information about their child´s care. Although a large majority of the parents were satisfied with their communication with doctors and nurses, only about half of the parents felt the nurses and doctors understood their emotional situation very well. Some parents would have desired easier access to conversations with doctors and wanted medical information to be given directly by doctors rather than by nurses. Parents’ communication with the staff was hampered when many different nurses were involved in caring for the infant or when the transfer of information in connection with shift changes or between the maternity ward and NICU was poor. Parents also desired to be present during doctors’ rounds on their infant.

**Conclusions:**

Training both doctors and nurses in communication skills, especially in how to meet parents’ emotional needs better, could make communication at the NICU more effective and improve parental well-being. Creating a framework for the parents of what to expect from NICU communication might also be helpful. In addition, our results support the use of primary nurse teams to improve continuity of care and thereby promote successful communication.

## Background

The parents of infants hospitalized in the neonatal intensive care unit (NICU) find themselves in a situation of emotional strain and potential crisis [1-5]. Being separated from their child is painful [4-7], the hospital environment is unfamiliar [1,5,8,9] and parents are dependent on doctors and nurses to be able to cope with their situation [10,11] and familiarize themselves with the care of their child [5,6,12]. Good communication between parents and staff is therefore an essential part of the support offered to parents in the NICU. As previous studies have shown, parents whose children are being cared for in the NICU feel the need not only to understand the technical aspects of care, but also to have opportunities to discuss their experiences and emotions with staff members [10,11]. Parents feel supported by emotional responsiveness to their needs on the part of staff [6,11,13-15] as well as by being continuously informed about their child’s state of health and treatment [5,9,10,16,17]. Parents’ need for communication, however, is not always met by the NICU staff [1,5,10,14,18,19], and staff may not be aware of communication problems in the same way as parents [14,20]. When parents feel dissatisfied with their communication with staff, their stress and anxiety increase [1,14,21] and they find it more difficult to establish a close relationship to their child [1,22,23].

Parent–provider communication in the NICU presents special challenges due to parental stress and the complexity of the highly technologized environment [6,10,24]. The purpose of communication between staff and parents in NICU is not only to inform the parents about their child’s medical condition and treatment; the doctors and nurses must educate the parents as well and invite them to participate in decision-making and the care of their child. The staff must use their communication skills to understand and support the parents in their emotional situation in NICU [25]. The purpose of the present study was to describe strengths and weaknesses in parent–nurse and parent–doctor communication, in order to improve our understanding of parents’ needs in this area.

## Methods

### Design

In order to describe different aspects of strengths and weaknesses in parent–nurse and parent–doctor communication in the NICU a survey instrument was used. Parents rated their experiences of communication in the NICU on a Likert scale and described their experiences in freely worded answers. The questions in the survey instrument were based on articles [10,11,25] delineating good communication in the NICU as satisfying the following conditions: doctors and nurses are available for conversations with the parents (questions 2 and 10); the parents’ questions are answered (questions 3 and 11) and these answers are easy for them to understand (questions 4 and 12); the instructions/information to the families are given in such a way that parents can understand their child’s medical condition and care and participate in decision-making concerning their child (questions 5 and 13); nurses and doctors understand the parents’ emotional situation (questions 6 and 14); finally, nurses and doctors encourage the parents to participate in the care of their infant (questions 7 and 15). In addition, the parents also rated how satisfied they were with their communication with nurses and doctors in general (questions 1 and 9). They were also asked whether they felt something had been missing in their communication with the staff (questions 8 and 16).

### Setting

The study was conducted over the course year at the Level III NICU [26] of a university hospital that treats about 1,000 newborns per year, including extremely premature and critically ill infants transported from other regional hospitals. The NICU had a high turnover of patients, often leading to a high workload for doctors and nurses. When the child’s medical condition was sufficiently stable, he/she was transferred to a level II neonatal unit or was discharged home. The NICU has 22 beds divided into four rooms (two intensive care rooms and two intermediate care rooms) and a staff of 120, including doctors, registered nurses and nursing assistants. During office hours 4–5 doctors, including 2–3 attendings, 1–2 neonatal fellows, and 0–1 pediatric residents, work at the NICU. Each doctor is on service for a period of two to three weeks. After making rounds at the NICU, the doctors make rounds at the normal newborn nurseries. They also work at the follow-up clinic, teach medical students, and have administrative and clinical meetings in the afternoons. This means that the number of physicians present at the unit is highly variable during office hours. From 5 p.m. to 8 a.m., one doctor is on call in-house at the NICU and another doctor is on call from home. Throughout the day, 5–6 registered nurses and 5–6 nursing assistants work at the NICU. The unit does not use primary registered nurse teams, neonatal nurse practitioners, or physician assistants.

### Participants

Parents of NICU patients were invited to participate in the study during the last days of their child’s stay in the NICU. Parents who did not understand or could not make themselves understood in Swedish were excluded. The researcher responsible for recruiting parents for the study visited the NICU on 80 occasions evenly distributed over the year. Out of a total of 442 parents, 44 non-Swedish-speakers were excluded. Both parents of the same family were given the possibility of filling in the questionnaire. Of the 398 parents asked to participate in the study, 20 declined and the remaining 378 parents gave their informed consent.

### Data collection

Parents were asked to complete a survey consisting of sixteen questions about their experiences of communication with nurses and doctors in the NICU respectively. In each question the parents evaluated some aspect of communication on a five- or six-point Likert scale. They also had the opportunity on each question to comment on their experiences in their own words.

To ensure that the questions were easy to understand, the survey instrument was tested through a pilot study in which 29 parents completed the survey in its entirety and were asked to critique its structure and wording.

The following data were collected on the infants: gestational age, birth weight, duration of stay in the NICU, number of newborns per family, destination after discharge from the NICU and (the last six months of the study) the sex of the parent answering the survey.

### Data analysis

Data were analyzed using IBM SPSS Statistics 20.0 [27]. Differences between parents’ answers to questions regarding nurses and doctors, and differences between mothers’ and fathers’ answers were compared using Friedman’s test [27]. Effect-size calculations were used to determine the clinical significance of the results. Based upon Cohen’s suggestions, effect sizes of 0.2 to 0.5 have been regarded as being small, those of 0.5 to 0.8 as moderate and those of 0.8 or above as large [28]. A level of significance of less than 0.05 was considered statistically significant.

The free-response answers to the survey questions were transferred verbatim to Microsoft Excel files and inductively analyzed by qualitative manifest content analysis as described by Graneheim and Lundman [29]. Elements in the participants’ descriptions of strengths and weaknesses in parent–nurse and parent–doctor communication were sorted into categories and subcategories within the two overarching domains designated as “strengths” and “weaknesses”. Within each domain, category and subcategory, each respondent was counted only once irrespective of his or her total number of comments in that area. When given the opportunity to describe their experiences in their own words, most parents mentioned both strengths and weaknesses that they had found in their communication with NICU staff.

## Results

### Characteristics of parents and infants

Of the 378 parents who received the survey, 270 (71.4%) completed it. Collectively they had a total of 169 children in the NICU, whose characteristics are presented in Table 1. Most infants (70.4%) were either moderately preterm or term. The length of stay in the NICU varied from 1 to 116 days, but the majority (65.3%) stayed at the hospital between three days and two weeks.

**Table 1:** Characteristics of infants whose parents participated in the study

**Variable**	**Number (%)**	**Median/ mean (SD)**	**Range**
**Gestational Age (weeks)**		35/35 (5.0)	23–43
Extremely preterm birth (<28 weeks)	23( 13.6)		
Moderate preterm birth (28+0 - 33 weeks)	27 (16.0)		
Preterm birth (33+1 - 37 weeks)	53 (31.4)		
Term birth (> 37 weeks)	66 (39.0)		
Total	169 (100)		
**Birth weight (grams)**		2463/2527 (1132)	535–5150
< 1000	20 (11.8)		
1000-1499	16 (9.5)		
1500-2499	49 (29.0)		
>2500	84 (49.7)		
Total	169 (100**)**		
**Length of stay in the NICU (days)**		6.0/14.9 (22.6)	1–116
1-2.5	23 (13.8)		
3-7.5	86 (51.5)		
8-14.5	23 (13.8)		
15-29.5	10 (6.0)		
> 30	25 (15.0)		
Total	167 (100)		
**Discharge to**			
Home without neonatal home care	53 (31.6)		
Home with neonatal home care	10 (6.0)		
Newborn Nursery	18 (10.7)		
NICU level II at other hospitals	79 (47.0)		
Pediatric ward at the same hospital	8 (4.8)		
Total	168 (100)		
**Number of families with singletons or multiples**			
Singletons	141 (91.0)		
Twins	13( 8.4)		
Triplets	1 (0.6)		
Total	155 (100)		

Both parents in families participating in the study were encouraged to answer the questionnaire separately. We collected information on the sex of the parents answering the questionnaire for the last 144 participants. Of these, 75 (52.0%) were mothers and 69 fathers (48.0%).

### Answers to the survey questions

Distributions of parents’ answers to survey questions about conversations with the NICU staff are shown in Table 2. Overall, parents rated communication in the NICU highly. A large majority of the parents stated that they were satisfied with their conversations with nurses and doctors and found it easy to communicate with nurses and doctors.

**Table 2:** Distributions of parents’ answers to survey questions about conversations with the NICU staff

**Survey questions**	**Answers**	**Number (%)**		**P-value**
		**Nurses**	**Doctors**	
1. How satisfied are you with the conversations you have had?	Very satisfied	190 (70.4)	167 (62.3)	0.03*
	Fairly satisfied	68 (25.2)	61 (22.8)	
	Neither satisfied nor dissatisfied	10 (3.7)	37 (13.8)	
	Fairly dissatisfied	1 (0.4)	2 (0.7)	
	Very dissatisfied	1 (0.4)	1 (0.4)	
	Total	270 (100)	268 (100)	
2. How easy has it been for you to communicate with nurses/doctors in the NICU?	Very easy	184 (68.4)	158 (59.2)	0.001*
	Fairly easy	74 (27.5)	75 (28.1)	
	Neither easy nor difficult	8 (3.0)	31 (11.6)	
	Fairly difficult	3 (1.1)	2 (0.7)	
	Very difficult	0 (0)	1 (0.4)	
	Total	269 (100)	267 (100)	
3. Have you received answers to your questions?	Yes	256 (95.2)	247 (92.5)	0.194
	No	13 (4.8)	20 (7.5)	
	Total	269 (100)	267 (100)	
4. Have the answers you have received been easy to understand?	Yes, always easy	114 (42.2)	143 (53.8)	0.071
	Yes, almost always easy	143 (53.0)	91 (34.2)	
	Neither easy nor difficult	8 (3.0)	22 (8.3)	
	No, not always easy	5 (1.9)	10 (3.8)	
	No, often not easy	0 (0)	0 (0)	
	No, almost never easy	0 (0)	0 (0)	
	Total	270 (100)	266 (100)	
5. Have the instructions/information that you have received about the care of your child been easy to understand?	Yes, always easy	151 (56.3)	155 (58.1)	0.253
	Yes, almost always easy	106 (39.6)	73 (27.3)	
	Neither easy nor difficult	6 (2.2)	31 (11.6)	
	No, not always easy	5 (1.9)	7 (2.6)	
	No, often not easy	0 (0)	1 (0.4)	
	No, almost never easy	0 (0)	0 (0)	
	Total	268 (100)	267 (100)	
6. How well do you feel that the staff you talked to in the NICU understood your emotional situation?	Very well	154 (57.0)	136 (51.5)	0.001*
	Fairly well	84 (31.1)	59 (22.3)	
	Neither well not poor	28 (10.4)	61 (23.1)	
	Not very well	3 (1.1)	5 (2.0)	
	Not at all well	1 (0.4)	3 (1.1)	
	Total	270 (100)	264 (100)	
7. Did you feel the NICU staff encouraged you to participate in caring for your child?	Very well	178 (66.2)	143 (54.2)	0.0001*
	Fairly well	69 (25.7)	35 (13.3)	
	Neither well not poor	16 (5.9)	79 (29.9)	
	Not very well	6 (2.2)	4 (1.5)	
	Not at all well	0 (0)	3 (1.1)	
	Total	269 (100)	264 (100)	
8. Was there something you felt was missing in communication with NICU staff?	Yes	73 (27.2)	56 (21.2)	0.101
	No	194 (72.4)	208 (78.8)	
	Total	267 (100)	264 (100)	

Friedman’s test was used to compare the distribution of answers regarding doctors and nurses. * Represents significant difference between the distribution of answers regarding doctors and nurses (p<0.05).

Almost all parents stated that they had received answers to the questions that they had from both nurses and doctors. In both cases, they felt that the answers were always or almost always easy to understand. Almost all parents also stated that the information and instructions that they had received were always or almost always easy to understand (95.9% for nurses, 85.4% for doctors).

Although a large majority of parents felt that the staff understood their emotional situation, only about half of the parents felt very well understood in this respect. Almost all the parents felt encouraged by the nursing staff to participate in the care of their infants; about 2/3 felt encouraged by doctors to do so.

In spite of mostly expressing satisfaction with various aspects of communication with NICU staff, many parents felt that something was lacking in communication with nurses (27.2%) and doctors (21.2%). The parents’ freely worded comments gave us important information about the reasons for this dissatisfaction (see Tables 3 and 4).

**Table 3:** Distributions of parents’ (n=226) descriptions of strengths and weaknesses of communication with nurses

**Domains**	**n (%)**	**Categories**	**n (%)**	**Subcategories**	**n (%)**
Strengths	204 (90.3)	Emotional support	180 (79.6)	Kind	67 (29.6)
				Understanding	52 (23.0)
				Took time	46 (20.3)
				Helpful	33 (14.6)
				Considerate	27 (11.9)
		Good information giving	131 (72.7)	Open and truthful	30 (13.2)
				Continuous information	28 (12.3)
				Gave answers to questions	28 (12.3)
				Gave good explanations	21 (9.3)
				Clear	20 (8.8)
		Professionalism	65 (28.7)	Competent	29 (12.8)
				Knowledgeable	29 (12.8)
				Confident	22 (9.7)
				Calm	18 (8.0)
				Took good care of the child	11 (4.9)
Weaknesses	118 (52.2)	Lack of emotional support	67 (29.6)	Lack of responsiveness	27 (11.9)
				Lack of focus on the parents' feelings	15 (6.6)
				Uninterested	13 (5.8)
				Unhelpful	11 (4.9)
				Unempathetic	10 (4.4)
		Poor information giving	76 (33.6)	Poor information	74 (32.7)
				Hard to get information	19 (8.4)
				Unclear	14 (6.2)
				No updates	14 (6.1)
				Different answers from different nurses	10 (4.4)
		Lack of professionalism	34 (15.0)	Incompetent	14 (6.2)
				Rude	13 (5.8)
				Seemed stressed	10 4.4)
				Left parent out of child's care	10 (4.4)
				Irritated	5 (2.2)
		Organizational problems	72 (31.9)	Lack of collaboration with maternity ward	38 (16.8)
				Lack of staff continuity	32 (14.2)
				Different attitudes of different nurses	23 (10.2)
				No nurse specially responsible for patient	10 (4.4)
				Poor information during shift changes	8 (3.5)

The number of parents who described their experiences of communication with nurses in their own words is reported. Each parent is counted only once for each domain, category and subcategory. The five most common subcategories are reported. n=number.

**Table 4:** Distributions of parents’ (n=206) descriptions of strengths and weaknesses of communication with doctors

**Domains**	**n (%)**	**Categories**	**n (%)**	**Subcategories**	**n (%)**
Strengths	133 (64.6)	Emotional support	87 (42.2)	Took time	27 (13.1)
				Nice	16 (7.8)
				Understanding	11 (5.3)
				Empathetic	9 (4.3)
				Listened	9 (4.3)
		Good information giving	111 (53.9)	Clear	65 (31.6)
				Gave good explanations	39 (18.9)
				Simple language	36 (17.5)
				Gave answers to questions	26 (12.6)
				Frequent, regular information	22 (10.9)
		Professionalism	22 (10.7)	Calm	17 (8.3)
				Knowledgeable	13 (6.3)
				Competent	11 (5.3)
				Confident	7 (3.4)
				Concerned about child's best	6 (2.9)
Weaknesses	153 (74.3)	Lack of emotional support	62 (30.1)	Didn’t take time	21(10.2)
				Lack of responsiveness	20 (9.7)
				Lack of focus on parents’ feelings	13 (6.3)
				Unempathetic	8 (3.9)
				Insensitive	7 (3.4)
		Poor information giving	100 (48.5)	Poor information	100 (48.5)
				Unclear	40 (19.4)
				Difficult language	24 (11.7)
				Information given via nurses	22 (10.7)
				Lack of continuous information	17 (8.3)
		Lack of professionalism	15 (7.3)	Seemed stressed	10 (4.9)
				Untruthful	5 (2.4)
				Insecure	3 (1.5)
				Awful	2 (1.0)
				Arrogant	1 (0.49)
		Organizational problems	72 (35.0)	Unavailability	71 (34.4)
				Lack of participation during rounds	18 (8.7)
				Different attitudes of different doctors	15 (7.3)
				Lack of staff continuity	13 (6.3)
				Lack of collaboration with maternity ward	8 (3.9)

The number of parents who described their experiences of communication with doctors in their own words is reported. Each parent is counted only once for each domain, category and subcategory. The five most common subcategories are reported. n=number.

The sex of the parent answering the survey was available for 70 couples. For each couple, the mother’s and father’s answers were compared to each other. No significant differences were found (p>0.05).

### Differences between nurses and doctors: effect size

Each parent’s answers to survey questions on communication with nurses were compared with the corresponding answers concerning doctors (Table 2). Significant differences were found in several areas (Table 2, p<0.05).

Effect-size calculations were used to determine whether the statistical differences that were observed between parental ratings of communication with nurses versus doctors were clinically meaningful. Table 5 shows the values from the survey questions used in the calculations and gives the results of the calculations. The results are based on the average from two means corrected for dependence between means using Morris and DeShon’s equation 8. The effect sizes for the differences as measured by SRM (standardized response mean) were all low, indicating that even though these differences were statistically significant their clinical significance as measured by Cohen [28] was slight.

**Table 5:** Results of the effect size calculations

**Survey questions**	**Pearson’s r coefficient**	**Effect size (Cohens’ d)**
1. How satisfied are you with the conversations you have had?	0.28	0.22
2. How easy has it been for you to communicate with nurses/doctors in the NICU?	0.33	0.23
6. How well do you feel that the staff you talked to in the NICU understood your emotional situation?	0.54	0.26
7. Did you feel the NICU staff encouraged you to participate in caring for your child?	0.28	0.37

*Effect sizes according to Cohen.

0.2-0.5=small.

0.5-0.8=moderate.

0.8 or above=large.

### Parents’ freely worded descriptions of their experiences

In the free-response part of the survey, 226 parents gave a total of 996 answers in their own words regarding their communication with nurses (median 5.3 comments per parent, range 1–8). Of 226 parents, 108 described only strengths, 22 only weaknesses and 96 both strengths and weaknesses in communication with nurses (Table 3). 206 parents described their communication with doctors in a total of 826 answers (median 4.0 comments per parent, range 1–8). 53 of these parents described only strengths, 73 only weaknesses and 80 both strengths and weaknesses in their communication with doctors (Table 4).

Qualitative manifest content analysis was used to classify these 1822 comments by 243 parents into two groups: those describing strengths in communication, with the subcategories of emotional support, good information giving and professionalism, and those describing weaknesses in communication, with the subcategories of lack of emotional support, poor information giving, lack of professionalism and organizational problems (Tables 3 and 4).

In order to highlight which specific strengths and weaknesses were perceived as most prevalent by the parents, we counted the number of parents who reported positive and negative experiences of communication with nurses/doctors in their own words. Each parent is counted only once for each domain, category and subcategory. The five most common subcategories are reported in Tables 3 and 4.

### Strengths and weaknesses in communication

#### Emotional support

Nurses and doctors who gave emotional support to the parents were described as considerate, empathetic or helpful, nice or kind; they took time, showed understanding and were considerate (Tables 3 and 4). A larger number of parents described having received emotional support from nurses than from doctors (Tables 3 and 4).

*“When I was sad and anxious they were there to listen or lay a hand on my shoulder”* (parent of an infant treated for hypoglycemia describing communication with nurses). *“We didn’t meet the doctors nearly as often as the nurses, so there wasn’t as much of an emotional relationship with the doctors”* (parent of a moderate premature infant describing communication with doctors).

Some parents felt that doctors had showed understanding of their emotional situation by referring to himself/herself. *“Doctor X told us that she had children herself”* (father of a premature infant treated for necrotizing enterocolitis describing communication with doctors).

Parents’ trust in the doctor’s assessment of the medical situation of their child helped them feel reassured. *“When I wasn’t sure whether my son would make it, I would ask Dr. X if he was worried, because if he felt they had everything under control, I wasn’t so worried anymore”* (parent of an extremely premature infant describing communication with doctors).

#### Lack of emotional support

Parents who felt that communication with NICU nurses and doctors had been marred by a lack of emotional support described the nurses and doctors as unempathetic, insensitive, or uninterested; they were felt to lack responsiveness or focus on the parents’ feelings (Tables 3 and 4). Several parents mentioned that doctors who did not give emotional support failed to take time to talk to parents (Table 4).

*“When I was tired and started to cry the response was just ‘don’t cry’ without any other comment or asking why I was crying. I felt they were fairly brusque and uninterested, maybe they thought our baby was too healthy compared to other babies in the unit”* (parent of an infant treated for infection describing communication with nurses). *“There was no time for anything but talking about the baby”* (mother of an infant treated for hypoglycemia describing communication with doctors). *“I don't feel that the doctors were so interested in understanding how we felt”* (parent of an infant treated for hypoglycemia describing communication with doctors).

Some parents described situations where they had felt that staff had not understood their emotional situation or met their need for support.

*“The staff is so used to seeing children with catheters and lines that they forget how hard it is on parents”* (parent of a moderate premature infant describing communication with nurses). *“When we were discharged we had a really hard time. Few staff members seemed to understand how hard it was and no one asked”* (parent of an infant treated for X syndrome describing communication with nurses). *“Only one doctor showed that she understood our situation, the others didn’t. It is terrible if they can’t or don’t have the energy to understand”* (parent of an infant treated for hypoxia describing communication with doctors).

Several parents stated that they were worried and needed emotional support although they were aware that their infant was not very ill.

*“Regardless of how sick the baby is, the parents have a hard time. Try to understand this and don’t rank us according to the severity of the baby’s condition”* (parent of an infant treated for icterus describing communication with nurses). *”I am a physician myself, but in the NICU I was, ”only a mother” and I was quite worried although our child was doing well. I would like to have vented my anxiety even if there was no real reason for it”* (mother of a moderate premature infant describing communication with doctors).

#### Good information giving

Many parents appreciated having received regular information and good explanations about their child’s care from both nurses and doctors and getting their questions answered (Tables 3 and 4). It made a difference to them when doctors gave them clear information, prepared them before examinations of their child and explained the results to them afterwards. Parents reported that communication with doctors was easier when the doctor used simple language (Table 4). Nurses’ readiness and openness in answering the questions parents had about their child’s condition made parents feel well-informed (Table 3).

*“They explained the procedures well and told us when they were changing something and why. We were also informed in advance about what they thought the next step would be”* (parent of an infant treated for hypoglycemia describing communication with doctors). *“Most of them were open to our questions and what might have been silly worries”* (parent of an extremely premature infant describing communication with nurses).

#### Poor information giving

Many parents stated that they had been given poor or unclear information by nurses and doctors (Tables 3 and 4). Some parents had received different information from different nurses. Both nurses and doctors were often stated to have provided too little information (Tables 3 and 4).

*“When I came up (to the NICU) one day after my C-section, no one took the time to explain what had happened. My daughter had been moved from a bed to an incubator and to CPAP treatment. SHOCK!”* (mother of a premature infant describing communication with nurses).

Some parents said doctors had used medical jargon that was difficult to understand (Table 4).

*“They throw terminology at you that you don’t understand and are sometimes totally insensitive to the fact that it feels like a death sentence for your child when they come in and explain test results and then rush out again before the information has sunk in”* (parent of an extremely premature infant describing communication with doctors). *“Too many medical terms and too many different answers to the same questions which makes you think they don’t really know what they’re talking about”* (parent of an extremely premature infant describing communication with doctors).

Some parents criticized the fact that they had received information via nurses rather than directly from the doctors (Table 4).

*“Information should not go through several people, the doctors tell the nurses things but it would be better if doctors informed the parents directly”* (parent of an extremely premature infant describing communication with nurses). *“Doctors communicated sometimes though the nurses and it felt that some information was lost”* (parent of an infant treated for infection describing communication with doctors).

Parents whose infants did not have severe diseases or stayed in the NICU for a short time felt that they did not have the possibility of talking with a doctor as much as they would have liked. However, these parents got the information they needed from the nurses instead.

*“I am happy with the conversations I had but had wanted to have more conversations with doctors. I got information from the nurses”* (parent of an infant treated for infection describing communication with doctors). *“It felt strange not to meet the doctors who make decisions about the treatment but it was OK because the nurses were good at communicating”* (mother of an infant treated for infection describing communication with doctors). *”I did not see the doctors much, but it was probably because our son ”behaved himself” and just needed to grow”* (parent of a moderate premature infant describing communication with doctors).

#### Professionalism

Nurses and doctors who were competent, calm, confident, and knowledgeable were felt to be professional (Tables 3 and 4).

*“I felt safe leaving my child with the staff”* (mother of moderate premature infant describing communication with nurses). *“They were focused and stayed calm… Their confidence gave me hope and strength”* (parent of an infant treated for hypoxia describing communication with doctors).

Parents praised the fact that nurses had encouraged them to participate in the care of their child, which gave them the sense of being understood on an emotional level. Some parents stated that they had not discussed their own involvement in their child’s care with doctors at all, but that this had been part of the nurses’ job.

*“They encouraged us to hold and nurse the child very soon after birth”* (mother of a moderate premature infant describing communication with nurses). *“I don’t feel the doctor was directly involved in this, it was more through the nurses. The doctor’s job was more to evaluate how my daughter was doing and her development”* (parent of a premature infant describing communication with doctors).

#### Lack of professionalism

Overall, parents seldom commented on lack of professionalism in their freely worded comments (Tables 3 and 4). Some parents mentioned that nurses were incompetent, rude or stressed (Table 3). Some parents felt that doctors were stressed (Table 4).

Some parents found it hard to participate in caring for their child when they were not given enough practical instructions or when nurses left them out of decisions concerning the care of their child. Instead they had a feeling of being unneeded by their child or in the way. Those parents who did not receive sufficient information and guidance from nurses sometimes reported feeling that they were being excluded and not given the chance to take care of their child.

*“As a parent you want to do as much as possible and be involved. It often felt as if he belonged to someone else”* (mother of an extremely premature infant describing communication with nurses).

Some parents felt the staff sometimes underestimated their abilities which they found offensive.

*“Since this wasn’t my first child some instructions could feel insulting, as if I did not know how to hold a baby. Sometimes I got instructions without wanting or needing them”* (mother of an infant treated for hypoglycemia, infection and icterus describing communication with nurses).

A few parents described situations where they felt it was best to follow the routines of the unit even if they as parents had another opinion.

*“There were some who made you feel uncomfortable if you didn’t follow their routines”* (parent of a moderate premature infant describing communication with nurses).

#### Organizational problems

Organizational factors reported most often by parents as impairing communication with nurses included lack of collaboration with maternity ward, lack of staff continuity, and different attitudes of different nurses and not having a nurse especially responsible for the parents (Table 3). For parent-doctor communication, unavailability of doctors was the problem reported most often, followed by lack of parent participation during rounds, different attitudes of different doctors, and lack of staff continuity (Table 4).

*“We lost our baby (twin) and had a meeting with the doctor about what had happened and his cell phone rang all the time, the staff wanted him. We felt really badly after the meeting, this shouldn’t happen in these kinds of meetings”* (mother of an extremely premature twins describing communication with doctors). *“The doctors have too many babies to take care of at a time. It would be better if there weren’t so many patients, but thanks so much for all you have done for us”* (father of an extremely premature infant describing communication with doctors).

Poor continuity of the staff caring for the infant and communication failures between staff members impaired communication with parents. Informing the parents of changes in staffing and introducing the new nurse beforehand to the family might improve the transition, if continuity of staffing is not a possibility

*“Continuity has been poor sometimes. It was hard to know who would be working when. Just when you were beginning to have confidence in someone they would go work somewhere else and not come back. I wish the staff had been better at letting us know when we would meet them next”* (parent of an extremely preterm infant describing communication with nurses).

Some parents reported that defects in communication between the NICU and the maternity ward or in conjunction with shift changes resulted in communication problems between nurses and parents (Tables 3 and 4). A lack of collaboration between different staff members or units sometimes meant that parents themselves felt responsible for informing staff concerning their child’s care.

*“At the normal newborn nursery they told me to pump manually, but when I came to the NICU they said no, it’s better to use the breast pump. Later when I went back to the newborn nursery they went on again about how I should pump manually and when I said they told me at the NICU to use the machine, they got angry”* (mother of a moderate premature infant describing communication with nurses).

Another organizational factor was the fact that the NICU consisted of four large rooms with several patients in each of them. In order to ensure patients’ privacy, parents were not generally allowed to be present during rounds. A feeling of being left out and insufficiently involved resulted when parents were not allowed to be present during rounds on their child.

*“In general I’d like to be there during rounds on my child. The same way adult patients are present when the doctors round”* (mother of an extremely premature infant describing communication with doctors). *“I really dislike the fact that they gave us very serious news concerning our child so that other parents could hear. It’s extremely important to talk to parents in a separate room”* (mother of a premature infant treated for necrotizing enterocolitis describing communication with nurses).

## Discussion

This large study consists of a quantitative and a qualitative analysis of strengths and weaknesses perceived by parents in their communication with doctors and nurses at the NICU. The quantitative analysis of parents’ answers to survey questions revealed that parents were overall satisfied with their conversations with both nurses and doctors. They felt that the information that they received from the health care professionals was easy to understand. The majority of the parents also felt that both doctors and nurses understood their emotional situation and encouraged them to participate in the care of their infant. Although statistical analysis revealed that nurses received higher ratings in these two areas, the parents were not specifically asked to compare doctors with nurses in the questionnaire. Moreover, effect size calculations indicated that the differences between nurses and doctors were not clinically significant. In addition, although parents’ communication with nurses and doctors overlaps to some extent, the professional groups are not in competition with each other. Instead, parents’ communication with the two professional groups can be seen as complementary. For example, doctors are mainly responsible for informing the parents about the baby’s medical condition and treatment, whereas nurses, who spend more time at the bedside, have more opportunities to support the parents emotionally and encourage them in their parental role.

It is well known from other studies about provider-patient communication that different patients have different information needs [10,11,25]. It is therefore also understandable that different NICU parents have different expectations about nurse-parent or doctor-parent communication. It is important for caregivers to ask the parents what they know and what questions they have.

The qualitative analysis of parents’ free-response answers to survey questions describes both strengths (emotional support, good information giving, professionalism) and weaknesses (lack of emotional support, poor information giving, lack of professionalism, organizational problems) of communication between parents and staff in the NICU. Parents describe their experience in communication with nurses and doctors most by positive terms, but at the same time they noted many flaws in specific aspects of communication, as revealed by their comments in the survey. For example, a mother whose child was treated for seven weeks in the NICU for prematurity stated that she was fairly satisfied with the communication she had had with the nurses but then wrote in her own words that two nurses had been inconsiderate and offensive. Another mother, whose term baby spent a month at the NICU for an uncommon illness also, stated that she was fairly satisfied but wrote that staff had not always taken time to explain things and conversations had been rushed. We might ask why these mothers did not rate themselves as fairly dissatisfied rather than fairly satisfied. An explanation for this apparent discrepancy may be that parents tend to evaluate NICU care in positive terms because they are generally grateful for the care their child has been given, as previous studies have shown [1,19]. Another possible explanation is that the answers to multiple-choice questions give an indication of parents' general impressions of communication in the NICU, while the free-response portion of the survey allowed them to focus on details of their experience. The use of complementary methods in communication research allowed a more nuanced picture to emerge than either method on its own would have [30].

This study confirms that, as several previous studies have shown, an empathetic attitude on the part of staff makes a difference to parents’ experience of communication in the NICU and to their relations with nurses [1,11,14] and doctors [10,31]. A good relationship between parents and staff, in turn, plays an important role in parents’ satisfaction with neonatal care [6,14,16] as well as their relationship to their child [12,23,32]. Several studies have focused on the ethical dimension of communication in the NICU [33-35]. Parents of NICU patients may feel helpless and vulnerable and are, to a certain degree, dependent on staff to be able to establish a physically and emotionally close relationship to their child. This can be seen as implying an ethical responsibility on the part of staff to do their best to help parents in this stressful situation [12].

Weiss et al. [17] have shown that availability of staff for conversations has a significant impact on parents’ perception of staff as empathetic and understanding. A lack of contact between staff and parents, on the other hand, can reinforce parents’ feelings of anxiety and exclusion. One probable reason why communication with nurses was described as a source of emotional support more often than communication with doctors is that nurses, because of the nature of their job, are more often physically present at the bedside and thus more available for emotional contact with families. The physical accessibility of nurses and the practical caregiving involved in their work may also explain why they were experienced as encouraging parents to be involved in caring for their child.

The NICU is staffed with more nurses than doctors per patient and the nurses have more opportunities to notice the needs of parents and to give them emotional support. But the number of nurses and shift changes can lead to problems in continuity. Lack of staff continuity was described by participants in the present survey as an impediment to effective information giving. Other studies have shown it to contribute to a sense of insecurity on the part of patients’ families [6,32].

The most important weakness in information-giving reported in this study was information that was too scanty or infrequent. Some parents complained that staff, especially doctors, used medical terminology that was hard to understand and that nurses gave information about their child’s medical condition. Parents need to be sufficiently informed about their child’s condition and treatment if they are to have confidence in the care their child is receiving and see their relationship to NICU staff as positive [5,10,12]. Parents who feel well-informed are satisfied with neonatal care [17,36] and feel involved in caring for their child [18,36,37].

In family-centered care (FCC), parents are kept well-informed and involved to a high degree in the care of their child and decisions affecting the child [38,39]. FCC has been shown to result in improved collaboration between parents and staff, decreased stress and insecurity on the part of parents [40], and their improved satisfaction with communication with doctors irrespective of the gravity of the child’s condition [37]. NICU staff can affect the degree to which care is family-centered by inviting parents to be involved in caring for their child or by failing to do so. The flaws in information giving reported by some parents in this study may be an indication that care is insufficiently family-centered.

The most pronounced organizational problem revealed by this study was doctors’ limited availability for discussions with parents. The parents wished to meet the doctor more often but understood that the doctors just did not have time for this. Kowalski et al. [31] have shown that parents of NICU patients, in particular those whose children are not gravely ill, feel they do not often meet the neonatologist. Nevertheless, parents were generally satisfied with their contacts with staff and trusted that the doctor would be available to talk to them if the child's condition worsened acutely. A study by Bramwell et al. [18] has shown that being present during rounds in the NICU enables parents to establish relationships with staff members and take part in discussions concerning their child. Parents in this study would have liked the opportunity of meeting the doctor during rounds.

One might expect that parents of very immature infants who have most medical problems and the longest duration of hospitalization would have more communication problems than fullterm infants with minor illnesses and a short hospitalization. However, analysis of our data stratified according to gestational age, birth weight, and duration of stay showed that the parents’ answers were not dependent on these variables. It seems that independent of the infant’s length of stay or birth weight or gestational age, parents are worried and stressed and need information and empathy from nurses and doctors as well as encouragement and support in their role as parents. Parents whose infants have most medical issues and a long length of stay also receive more attention and have more opportunities of communicating with the medical providers than parents of infants with minor illnesses. In addition, the background, previous experiences, and personality of the parents are likely to have an influence on their need for communication independent of the severity of the infant’s illness or the length of stay or gestational age.

Results from this single-center study cannot be extrapolated to other centers with differently organized doctor-nurse teams. For example, the Swedish health care system does not include intermediate medical providers such as Advanced Practice Nurses (Neonatal Nurse Practitioners) or Physician Assistants, who are an essential part of the NICU team in some other countries, such as the United States. Another weakness of this study was that we excluded parents who did not speak or understand Swedish. We always use interpreters in our NICU when communicating with such families. Another study would be needed to address the communication needs and problems of these families.

## Conclusions

This study can help the staff in the NICU to understand parents’ experiences of communication better and thus form a basis for improving communication between parent and staff. Although a large majority of the parents were satisfied with their communication with doctors and nurses, only about half of the parents felt the nurses and doctors understood their emotional situation very well. Training both doctors and nurses in communication skills, especially in how better to meet parents’ emotional needs, could make communication at the NICU more effective and improve parental well-being. Creating a framework for the parents of what to expect from communication in the NICU might also be helpful. In addition, our results support the use of primary nurse teams to improve continuity of care and thereby promote successful communication.

## Abbreviations

NICU: Neonatal Intensive Care Unit; FCC: Family centered-care.

## Competing interest

The authors report no conflict of interest.

## Authors’ contributions

HW and KB contributed to the conception and design of the study. HW and MBD performed the data collection and the data analysis. HW, MBD and KB interpreted the data and wrote the manuscript. All the authors read and approved the final manuscript.

## Authors’ information

Helena Wigert is Senior Lecturer at the Institute of Health and Care Sciences, The Sahlgrenska Academy at University of Gothenburg and at the Division of Neonatology, Sahlgrenska University Hospital, Gothenburg, Sweden.

Michaela Blom Dellenmark is a Specialist Nurse in Pediatric Nursing and Manager of Health Care Improvement at the Division of Pediatric Emergency Care and Pediatric Surgery, Sahlgrenska University Hospital, Gothenburg, Sweden.

Kristina Bry is an attending Neonatologist at the Sahlgrenska University Hospital and Professor of Neonatology at the University of Gothenburg, Sweden.

## Pre-publication history

The pre-publication history for this paper can be accessed here:

http://www.biomedcentral.com/1471-2431/13/71/prepub
